# Effects of vasopressin on anesthetic response time and circulatory dynamics of lidocaine

**DOI:** 10.1007/s10266-020-00585-x

**Published:** 2021-01-15

**Authors:** Shoko Fujimori, Katsuhisa Sunada

**Affiliations:** Department of Dental Anesthesiology, The Nippon Dental University School of Life Dentistry at Tokyo, 1-9-20, Fujimi, Chiyoda-ku, Tokyo 102-8159 Japan

**Keywords:** Anesthesia response time, Circulatory dynamics, Lidocaine, Rat, Vasopressin

## Abstract

This study aimed to investigate the hypothesis that vasopressin extends the anesthetic response time of lidocaine and does not affect the circulatory dynamics. Rats were sedated with isoflurane; subsequently, breathing was maintained through mechanical ventilation. We infiltrated the first molar area of the upper left jaw with saline (NS, test solution), 2% lidocaine (L), 0.025 IU vasopressin-supplemented 2% lidocaine, 0.05 IU vasopressin-supplemented 2% lidocaine, 0.1 IU vasopressin-supplemented 2% lidocaine, and 0.2 IU vasopressin-supplemented 2% lidocaine (VL_4_). Further, anesthetic response times were measured up to 30 min using electric pulp testing methods (*n* = 4). The anesthetic response times of NS, L, and VL_4_ were measured up to 45 min with the aforementioned results as reference values (*n* = 7). The circulatory dynamics of NS, L, VL_4_, and 0.2 IU vasopressin (V) were measured up to 45 min using a non-invasive blood pressure measuring device. VL_4_ extended the anesthetic response times of lidocaine compared to L (*p* < 0.05). Further, V and VL_4_ significantly increased the systolic and diastolic blood pressure and significantly decreased the pulse rate (*p* < 0.05). VL_4_ is not a suitable addition to the local anesthetic solution used in dentistry. Further study is needed to determine vasopressin concentration that extends the anesthetic effect without affecting the circulatory dynamics.

## Introduction

Lidocaine, a local anesthetic, is widely used in dental treatment due to its high tissue penetration and rapid onset of action. Further, its anesthetic effects disappear quickly due to its strong peripheral vasodilator actions and rapid absorption into the blood [1, 2]. Therefore, it is supplemented with epinephrine, which binds to catecholamine *α*_1_-receptors and constricts blood vessels. However, the vasoconstrictive effects of epinephrine are potent and may account for unexpected increases in blood pressure. Moreover, epinephrine acts on *β*_1_-receptors to increase cardiac contractions and the pulse rate, causing abnormal hypertension, angina pectoris, and myocardial infarction when administered to patients with cardiovascular diseases. Therefore, strict restrictions are placed regarding the epinephrine-supplemented lidocaine dose in patients with hypertension and ischemic heart disease [3–6]. Felypressin, which is used for local anesthesia as a vasoconstrictor similar to epinephrine, is a synthetic hormone compound where the tyrosine of vasopressin is replaced with phenylalanine. Similar to vasopressin, it acts directly on vascular smooth muscle, mainly the venous peripheral vessels, for constriction [5, 7]. The vasoconstrictive effects of felypressin depend on V1a receptors, and it does not bind to catecholamine receptors [7]. Therefore, it does not induce *β*_1_ receptor-stimulated cardiac hyperfunction. Consequently, it has a small effect on blood pressure and is relatively safe for hypertensive patients [8, 9].

Therefore, we believe that adding vasopressin to lidocaine as a vasoconstrictor, similar to felypressin, could allow safe local dental anesthesia in patients with cardiovascular disease. A rat study on somatosensory evoked potentials conducted by Murata et al. reported that vasopressin extended the duration of lidocaine’s action [10]. However, somatosensory evoked potentials represent electroencephalographic changes after electric stimulation, and it is unclear whether they measure biological responses to painful stimuli. Measurements with more clinical indicators are needed to determine the clinical utility of vasopressin-supplemented lidocaine. Electric pulp testing methods are widely used as clinical perception tests for dental pulp and are widely used to assess anesthetic effects [11–14]. Therefore, this study used electric pulp testing methods to measure anesthetic effects. Numerous reports regarding the effects of vasopressin on circulatory dynamics suggest that a large vasopressin dose causes hypertension, bradycardia, and cardiac arrest [15–17]. Therefore, the effects of vasopressin on circulatory dynamics should be examined. This study aimed to investigate the hypothesis that vasopressin extends the anesthetic response time of lidocaine and has no detrimental effect on circulatory dynamics.

## Materials and methods

This study was approved by the Animal Experiment Committee of the Nippon Dental University School of Life Dentistry (approval number 17-14-4) and was conducted following the committee regulations.

Male Wistar rats weighing 300–400 g were placed in anesthetic boxes filled with 5% isoflurane vaporized gas. Subsequently, they were removed after immobilization and orotracheally intubated. A Hashimoto-style opener (Nonaka Rikaki Co., Ltd., Tokyo, Japan) was attached, and a ventilator was connected to the tracheal tube. Subsequently, respiratory care was performed through mechanical ventilation. The ventilation rate was set at 70 breaths per min, while the tidal volume was set at 3.0 mL. We used 0.3–1.0% isoflurane for anesthetic maintenance. Body temperature was maintained at 37 °C using a warming device.

### Determination of vasopressin concentration

We confirmed the response to stimuli before test-drug administration as follows. An electric pulp testing device (PULP TESTER^®^, YOSHIDA DENTAL TRADE DISTRIBUTION Co., Ltd., Tokyo, Japan) was used to create counter electrode clips and probes. Using paste, the counter electrode clips and probe tip were placed in contact with the left anterior limb of the rats and the occlusal surface of the test teeth (Cardio Cream^®^; NIHON KOHDEN Co., Ltd., Tokyo, Japan). The target teeth were the first molars on the upper-left jaw, as previously described by Murata et al. [10]. Based on the study by Serpe et al., the intensities in the electric pulp testing device were increased over 30 s to a maximal stimulation of 80. Subsequently, rats showing pain responses (extremity movements other than the whiskers, head, and left foreleg) were considered positives. We excluded rats that did not show a pain response [11].

After confirming the pain response, the test-drug concentration that extended the anesthesia time was determined as follows. The palatal mucosa 2 mm from the mesial marginal gingiva of the first molar in the upper-left jaw was infiltrated with 50 μL of the test drug using a microsyringe with a 31G compatible needle (Ito Micro syringe^®^, ITO SEISAKUSHO Co., Ltd., Shizuoka, Japan). The following six types of test drugs were assessed, including normal saline (*n* = 4):

1. L: 2% lidocaine: [50 μL solution comprising a mixture of 25 μL of 4% lidocaine (Xylocaine^®^ Solution 4%, Aspen Japan Co, Ltd., Tokyo, Japan) and 25 μL of normal saline (OTSUKA NORMAL SALINE^®^, Otsuka Pharmaceutical Factory, Co., Ltd., Tokushima, Japan)]
2. VL_1_: 2% lidocaine with 0.025 IU vasopressin: [50 μL solution comprising a 25 μL solution of 1.0 mL of vasopressin (PITRESSIN^®^INJECTION, DAIICHI SANKYO Co., Ltd., Tokyo, Japan) diluted with 19.0 mL of normal saline (1.0 units/mL of vasopressin) and 25 μL of 4% lidocaine]
3. VL_2_:2% lidocaine with 0.05 IU vasopressin: [50 μL solution comprising 25 μL solution of 1.0 mL of vasopressin diluted with 9.0 mL of normal saline (2.0 units/mL) and 25 μL of 4% lidocaine]
4. VL_3_: 2% lidocaine with 0.1 IU vasopressin: [50 μL solution comprising 25 μL solution of 1.0 mL of vasopressin diluted with 4.0 mL of normal saline (4.0 units/mL) and 25 μL of 4% lidocaine]
5. VL_4_: 2% lidocaine with 0.2 IU vasopressin: [50 μL solution comprising 25 μL solution of 1.0 mL of vasopressin diluted with 1.5 mL of normal saline (8.0 units/mL) and 25μL of 4% lidocaine]
6. NS: [50 μL of normal saline]

Pain responses were examined at 2, 4, 10, 15, 20, 25, and 30 min after the drug administration. Stimulation was repeated if a pain response was observed. Further, anesthesia loss was determined in case a pain response was observed on two consecutive occasions. The time between administering the test drug and loss of anesthesia was defined as the anesthetic response time.

### Measurement of the anesthesia time

The anesthetic response times of L and VL_4_ were measured based on the results obtained with the method mentioned above. Using the protocol mentioned above, measurements were made at 2, 4, and 10 min after administering the test drug and subsequently at 5-min intervals up to 45 min (*n* = 7).

### Measurement of circulatory dynamics

We measured the pre-and post-treatment circulatory dynamics by measuring the blood pressure and pulse rate using a non-invasive blood pressure measuring device (Softron^®^ BP98A-L; Softron Co., Ltd., Tokyo, Japan) in NS, L, VL4, and 0.2 IU vasopressin (V: 50 μL of a solution of 1.0 mL solution of vasopressin diluted with 4.0 mL of saline). Circulatory dynamics before local anesthesia were measured in triplicate, with the mean being used as the baseline. Measurements were obtained at 2, 4, and 10 min after drug administration and subsequently at 5-min intervals up to 45 min (*n* = 7).

### Statistical processing

We used the unpaired Bonferroni t-test to analyze the anesthetic time. Comparisons regarding circulatory dynamics were determined using a two-way analysis of variance for circulatory dynamics. Accordingly, the paired and unpaired Bonferroni t-tests were used for intra- and inter-group comparisons, respectively.

## Results

### Determination of vasopressin concentration

**Fig. 1:**
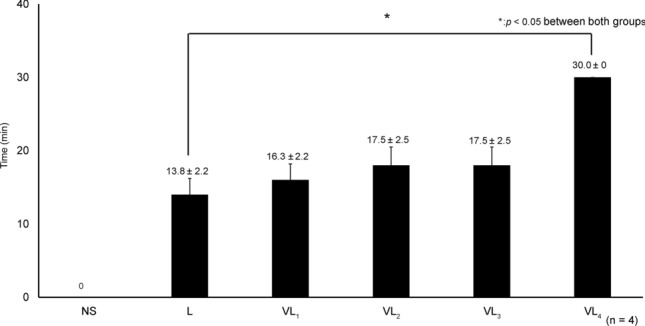
Vasopressin concentrations and anesthetic response time. NS: normal saline, L: 2% lidocaine, VL_1_: 2% lidocaine with 0.025 IU of vasopressin, VL_2_: 2% lidocaine with 0.05 IU of vasopressin, VL_3_: 2% lidocaine with 0.1 IU of vasopressin, VL_4_: 2% lidocaine with 0.2 IU of vasopressin. Anesthetic response time was significantly extended in VL_4_ compared to L (*p* < 0.0002)

For L, anesthetic effects disappeared after 20 min. For VL_1_, VL_2_, and VL_3_, but not for VL_4_, the anesthetic effects disappeared after 30 min (Fig. 1).

### Measurement of anesthetic response time

**Fig. 2:**
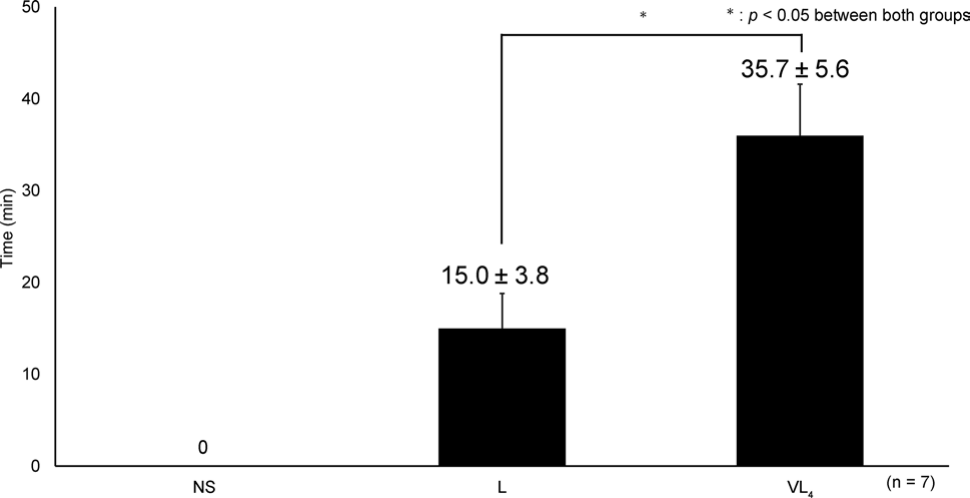
Comparison of anesthetic response time. NS: normal saline, L: 2% lidocaine, VL_4_: 2% lidocaine with 0.2 IU of vasopressin, A significant difference (*p* < 0.0001) was observed between L and VL_4_

Compared with L, VL_4_ showed a significantly extended anesthetic response time (Fig. 2).

### Measurement of circulatory dynamics

**Fig. 3:**
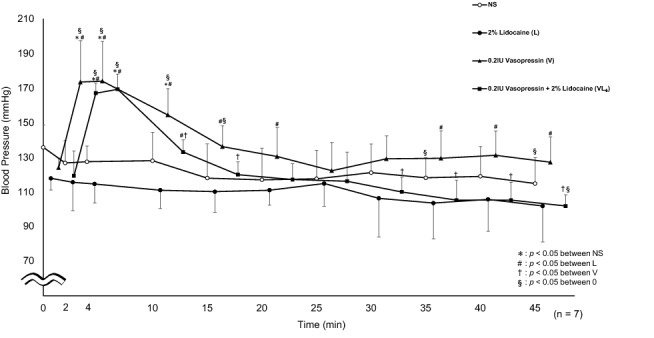
Systolic blood pressure. Significantly higher values were seen in VL_4_ at 2, 4, and 10 min (*p* < 0.0001, *p* < 0.0001, *p* = 0.0011, respectively) compared to L. VL_4_ was significantly elevated at 2, 4, and 45 min (*p* < 0.0001, *p* < 0.0001, *p* = 0.0192, respectively) compared to 0 min

Compared with L, VL_4_ showed significantly higher systolic blood pressure at 2, 4, and 10 min. Further, compared with the baseline value (0 min), VL_4_ showed a significant increase in systolic blood pressure at 2, 4, and 45 min (Fig. 3).

**Fig. 4:**
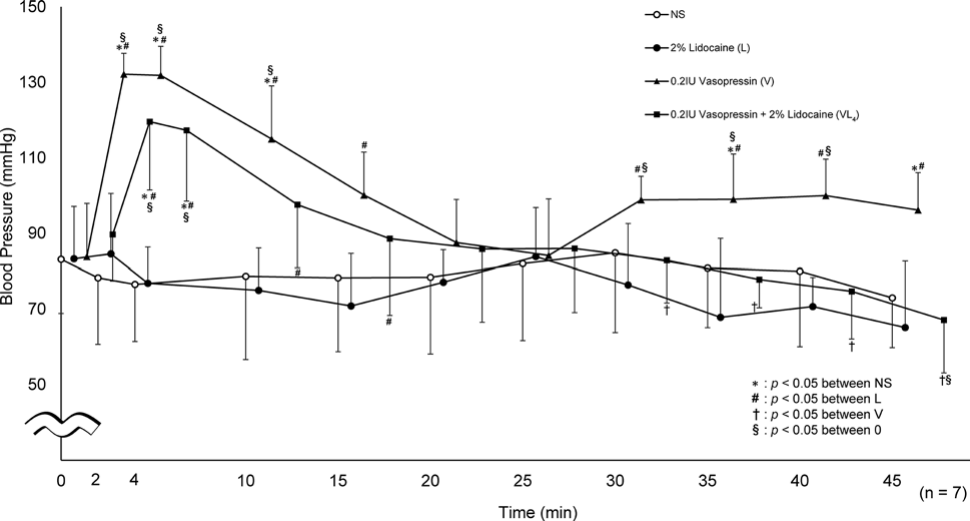
Diastolic blood pressure. Significantly higher values were seen in VL_4_ at 2, 4, 10, and 15 min (*p* < 0.0001, *p* < 0.0001, *p* = 0.0095, *p* = 0.0323, respectively) compared to L. VL_4_ was significantly elevated at 2, 4, and 45 min (*p* = 0.0004, *p* = 0.0012, *p* = 0.0074, respectively) compared to 0 min

Similarly, compared with L, VL_4_ showed significantly higher diastolic blood pressure at 2, 4, 10, and 15 min. Further, compared with the baseline value (0 min), VL_4_ showed a significant increase in diastolic blood pressure at 2, 4, and 45 min (Fig. 4).

**Fig. 5:**
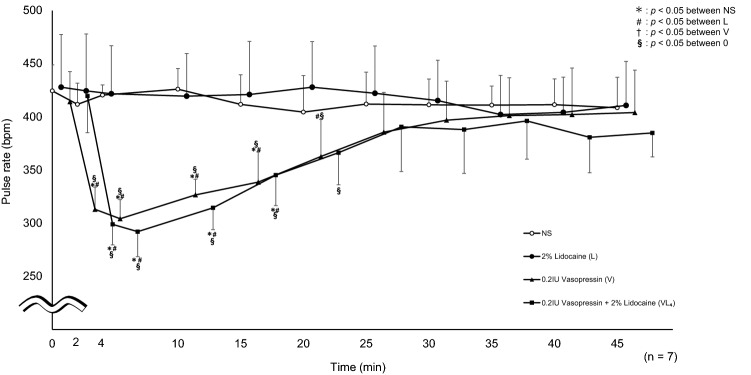
Pulse rate. Significantly lower values were seen in VL_4_ at 2, 4, 10, and 15 min (*p* = 0.0002, *p* < 0.0001, *p* < 0.0001, *p* = 0.0073, respectively) compared to L. VL_4_ was significantly reduced at 2, 4, 10, 15, 20 min (*p* < 0.0001, *p* < 0.0001, *p* < 0.0001, *p* = 0.0015, *p* = 0.0147, respectively) compared to 0 min

Compared with L, VL_4_ showed significantly lower pulse rates at 2, 4, 10, and 15 min. Compared with the baseline value (0 min), VL_4_ showed significantly lower pulse rates at 2, 4, 10, 15, and 20 min (Fig. 5).

## Discussion

We found that administering 2% lidocaine supplemented with ≦ 0.1 IU of vasopressin did not extend the anesthetic response time in rats. Nonetheless, the anesthetic response time was extended by administering 2% lidocaine with a 0.2 IU of vasopressin; however, this increased the systolic/diastolic blood pressure and decreased the pulse rate.

Lidocaine has a strong vasodilatory effect and is rapidly absorbed into the blood, which leads to the disappearance of the anesthetic effect [1, 2]. This phenomenon is prominent in the oral mucosa with abundant blood flow. Therefore, epinephrine, which has vasoconstrictive effects stronger than the vasodilatory effects of lidocaine, is used to supplement local dental anesthetics administered in such mucosa for sufficient anesthetic time. However, in addition to its vasoconstrictive effects on catecholamine-*α*_1_ receptors, epinephrine acts on *β*_1_ receptors to increase myocardial contraction and heart rate. Therefore, administering 36–54 μg epinephrine (about 2–3 dental cartridges) is recommended for patients with hypertension [6]. Felypressin, a synthetic polypeptide with a vasopressin-like amino acid sequence, has strong vasoconstrictive actions and does not act on the myocardial or conduction system. Therefore, it is clinically used as a highly safe vasoconstrictive drug for cardiovascular diseases [18]. However, few reports mention the application of local anesthetics supplemented with vasopressin in the dental fields. Middlehurst et al. reported that adding epinephrine and vasopressin to 2% lidocaine reduced epinephrine levels and improved catecholamine-derived arrhythmia risks [19]. Murata et al. reported that the anesthetic efficacy of lidocaine was extended by administering 0.03 IU/mL vasopressin indexed to somatosensory evoked potentials [10]. Contrastingly, we found that ≦ 0.1 IU vasopressin did not extend the anesthetic effects of lidocaine. This inconsistency could be attributed to the following reasons. Murata et al. used somatosensory evoked potentials to measure the anesthetic efficacy, which allows quantitative analysis of neural function; however, artifact contamination and amplitude changes could occur due to the small signal-to-noise ratio [20–23]. Moreover, somatosensory evoked potentials cannot measure responses associated with pure pain, including activation of Aδ and C fibers (which transmit pain) and Aα and Aβ fibers (associated with touch, vibration, and proprioception) and measurement of changes in the potentials of the central nervous system to stimuli [23, 24]. The electric pulp testing method is used to measure the pain threshold of the dental pulp by applying a weak current to the teeth and measuring the positive responses of the head and limbs. Serpe et al. administered an anesthetic solution to the mandibular foramen of rats and performed electric pulp tests to measure the anesthetic efficacy after an inferior alveolar nerve block [11]. Cho et al. evaluated the success rate of pulpal anesthesia using electric pulp tests to assess the correlation between the success rate of soft tissue anesthesia and pulpal anesthesia after inferior alveolar nerve block in healthy individuals [12]. Vongsavan et al. locally administered 4% articaine supplemented with 1:100,000 epinephrine to the mandibular first molars and canines in healthy volunteers and used electric pulp testing methods to measure anesthetic efficacy [13]. Furthermore, Oliveira et al. evaluated the anesthetic efficacy of needle-free jet injection and conventional injection needle insertion in volunteers requiring restoration of the maxillary first molars using an electric pulp testing method [14]. The findings mentioned above indicated that the electric pulp testing method could be reliable for measuring anesthetic effects. Electric pulp testing requires conductive media to allow maximal current flow from the electrode to the tooth surface [25, 26]. Further, there should be contact with the counter electrode to ensure a current circuit flowing from the tooth surface in vivo. However, we did not employ electric pulp tests designed for rats. Therefore, we made measurements using copper wires to create counter electrode clips and stimulation probes for rats. Overall, our findings may reflect clinical anesthetic actions better than measurement results using electroencephalographic readings as an indicator.

The VL_4_ vasopressin concentration that showed extended anesthetic action was 4.0 IU/mL (1 μL = 1/1000 mL, 0.2 IU / 50 μL = 0.2 IU × (1000/50) mL = 4.0 IU/mL. Guhl et al. reported that the vasoconstrictive effect of felypressin was about 5 times that of vasopressin [27]; therefore, 4.0 IU/mL of vasopressin corresponds to 0.8 IU/mL of felypressin. However, the felypressin level widely used as a local dental anesthetic is 0.03 IU/mL, denoting a large difference compared with VL_4_. The underlying reasons remain unclear; however, some possibilities have been proposed. For example, prilocaine supplemented with felypressin could have a weaker vasodilatory effect than lidocaine. Furthermore, the concentration of lidocaine and prilocaine were 2% and 3%, respectively. Moreover, the anesthetic effect of 3% prilocaine supplemented with 0.03 IU/mL felypressin has been reported to be insufficient [28].

Although VL_4_ extended the time of the anesthetic effects, it increased the blood pressure. The duration of anesthetic action is dependent on the vasopressin concentration at the administration site since it is affected by the local blood flow. However, the effects on circulation are caused by the administered vasopressin's action on peripheral blood vessels throughout the body via the bloodstream. Therefore, the total vasopressin dose should be considered. With an assumed bodyweight of 300 g for rats and 60 kg for humans, VL_4_ corresponds to administering 60/0.3 × 0.2 IU = 40 IU of vasopressin to humans. Butala et al. reported that 20 IU of vasopressin induced bradycardia, severe vasospasms, and hypertension in humans [15]. Furthermore, Lee et al. reported that a total vasopressin dose > 5.0 IU induced bradycardia and cardiac arrest [16]. Moreover, Fabin et al. reported that a single vasopressin dose should not exceed 2.5 IU [17]. Taken together, these reports demonstrate the difficulty in administering lidocaine supplemented with the abovementioned vasopressin dose to humans, even considering interspecies and administration-site differences.

Vasopressin constricts vascular smooth muscles on the venous side; however, high vasopressin concentrations constrict blood vessels on the arterial side, which increases blood pressure. Elevated blood vasopressin concentrations exert vasoconstrictor effects and increase the blood pressure. These changes activate baroreceptors in the aortic arch and carotid sinus and induce sympathetic inhibitory reflexes in the solitary nucleus, reflexively increasing parasympathetic tone, thereby causing bradycardia. [5, 7, 15, 16, 29]. Since vasopressin does not affect the conduction system, the observed bradycardia could be considered a baroreceptor reflex associated with elevated blood pressure. Given that the reported half-life of vasopressin in the blood is 10–20 min [17, 30–32], the 10- to 15-min increase in blood pressure and the associated bradycardia could have resulted from arterial contraction induced by the high vasopressin concentration and the resulting baroreceptor reflex.

This study has several limitations. First, we measured the effects on anesthetic action and circulatory dynamics under isoflurane inhalation. Under similar conditions, the vasopressin effects have been compared with those of lidocaine without vasopressin. However, the vasopressin effect on extending anesthetic actions and circulatory dynamics could differ from that of vasopressin administered to awake individuals. Second, the bodyweight of rats used in this study ranged from 300 to 400 g. Moreover, if the concentration of isoflurane is too high, rats would not respond to electrical stimulation. For these reasons, it was difficult to maintain anesthesia with isoflurane at the same concentration, and we used different concentrations depending on each rat. Therefore, it cannot be ruled out that the sedation level may have been different between the rats. Hence, further research is warranted.

In conclusion, administering 50 μL of 2% lidocaine supplemented with 0.2 IU of vasopressin into the oral cavity of rats extended the action duration of lidocaine; however, it increased the blood pressure and decreased the pulse rate. Further study is needed to determine vasopressin concentration that extends the anesthetic effect without affecting the circulatory dynamics.

## References

[1] Laurence W, Benjamin P, Chong T, Mehrdad N (2015). Pharmacokinetics and pharmacodynamics of lignocaine: a review. World J Anesthesiol.

[2] Gudin J, Nalamachu S (2020). Utility of lidocaine as a topical analgesic and improvements in patch delivery systems. Postgrad Med.

[3] Abraham-Inpijn L, Borgmeijer-Hoelen A, Gortzak RA (1988). Changes in blood pressure, heart rate, and electrocardiogram during dental treatment with use of local anesthesia. J Am Dent Assoc.

[4] Mehdi S, Mostafa S, Nafiseh E, Mohammad HN, Hossein S, Mehrdad S, Hamid RF, Shahrokh G (2013). The evaluation of perioperative safety of local anesthesia with lidocaine containing epinephrine in patients with ischemic heart disease. Acta Medica Iran.

[5] John AY (1995). Vasoconstrictor agents for local anesthesia. Anesth Prog.

[6] Herman WW, Konzelman JL, Prisant LM (2004). New national guidelines on hypertension: a summary for dentistry. J Am Dent Assoc.

[7] Rodrigo C, Laurival ADL, José R (2006). Cardiovascular effects of felypressin. Anesth Prog.

[8] Pelletier JS, Dicken B, Bigam D, Cheung PY (2014). Cardiac effects of vasopressin. J Cardiovasc Pharmacol.

[9] Sunada K, Nakamura K, Yamashiro M, Sumitomo M, Furuya H (1996). Clinically safe dosage of felypressin for patients with essential hypertension. Anesth Prog.

[10] Murata N, Sunada K, Hashimoto S (2020). Effect of adding vasopressin on the distribution of lidocaine in tissues, anesthetic action, and circulatory dynamic. Odontology.

[11] Serpe L, Franz-Montan M, dos Santos CP, da Silva CB, Nolasco FP, Caldas CS, Volpato MC, de Paula E, Groppo FC (2014). Anaesthetic efficacy of bupivacaine 2-hydroxypropyl-β-cyclodextrin for dental anesthesia after inferior alveolar nerve block in rats. Br J Oral Maxillofac Surg.

[12] Cho SY, Choi W, Kim J, Kim ST, Kim HJ, Jung IY (2019). Anesthetic efficacy of an inferior alveolar nerve block in soft tissue and correlation between soft tissue and pulpal anesthesia. Clin Oral Investig.

[13] Vongsavan K, Samdrup T, Kijsamanmith K, Rirattanapong P, Vongsavan N (2019). The effect of intraosseous local anesthesia of 4% articaine with 1:100,000 epinephrine on pulpal blood flow and pulpal anesthesia of mandibular molars and canines. Clin Oral Investig.

[14] Oliveira ACA, Amorim KS, Nascimento Júnior EMD, Duarte ACB, Groppo FC, Takeshita WM, Souza LMA (2019). Assessment of anesthetic properties and pain during needleless jet injection anesthesia: a randomized clinical trial. J Appl Oral Sci.

[15] Bina PB, Veena RS, Beena KP, Jayaprakash J, Jasmita K (2014). Bradycardia and severe vasospasm caused by intramyometrial injection of vasopressin during myomectomy. Saudi J Anaesth..

[16] Lee GG, Baek SY, Woo Kim T, Jeong CY, Ryu KH, Park DH (2018). Cardiac arrest caused by intramyometrial injection of vasopressin during a robotic-assisted laparoscopic myomectomy. J Int Med Res.

[17] Fabian M, Forsling ML, Jones JJ, Pryor JS (1969). The clearance and antidiuretic potency of neurohypophysial hormones in man, and their plasma binding and stability. J Physiol.

[18] Berde B, Weidmann H, Cerletti A (1961). On phenylalanine-2-lysine-vasopressin. Helv Physiol Pharmacol Acta.

[19] Middlehurst RJ, Gibbs A, Walton G (1999). Cardiovascular risk: the safety of local anesthesia, vasoconstrictors, and sedation in heart disease. Anesth Prog.

[20] Harrison SA, Lovely DF (1995). Identification of noise sources in surface recording of spinal somatosensory evoked potentials. Med Biol Eng Comput.

[21] Kalkman CJ, Romijn K, Denslagen W (1991). Eliminating diathermy-induced artifacts during intraoperative monitoring of somatosensory-evoked potentials: a hardware solution. J Clin Monit.

[22] Parsa V, Parker PA (1994). Multireference adaptive noise cancellation applied to somatosensory evoked potentials. IEEE Trans Biomed Eng.

[23] Kakigi R, Watanabe S, Yamasaki H (2000). Pain-related somatosensory evoked potentials. J Clin Neurophysiol.

[24] Rustamov N, Tessier J, Provencher B, Lehmann A, Piché M (2016). Inhibitory effects of heterotopic noxious counter-stimulation on perception and brain activity related to Aβ-fibre activation. Eur J Neurosci.

[25] Michaelson RE, Seidberg BH, Guttuso J (1975). An in vivo evaluation of interface media used with the electric pulp tester. J Am Dent Assoc.

[26] Cooley RL, Robison SF (1980). Variables associated with electric pulp testing. Oral Surg Oral Pathol.

[27] Guhl U (1961). Antidiuretic and pressor effects of arginine-8-vasopressin, lysine-8-vasopressin, phenylalanine-2-lysine-8-vasopressin in Man. Schweiz Med Wochenschr.

[28] Hirota Y, Sugiyama K, Joh S, Kiyomitsu Y (1986). An echocardiographic study of patients with cardiovascular disease during dental treatment using local anesthesia. J Oral Maxillofac Surg.

[29] Berling C (1966). Octapressin as a vasoconstrictor in dental plexus anesthesia. Odontol Revy.

[30] Yosten GL, Samson WK (2012). Pressor doses of vasopressin result in only transient elevations in plasma peptide levels. Peptides.

[31] Stoiser B, Mörtl D, Hülsmann M, Berger R, Struck J, Morgenthaler NG, Bergmann A, Pacher R (2006). Copeptin, a fragment of the vasopressin precursor, as a novel predictor of outcome in heart failure. Eur J Clin Invest.

[32] Treschan TA, Peters J (2006). The vasopressin system: physiology and clinical strategies. Anesthesiology.

