# Correction to: Effects of vasopressin on anesthetic response time and circulatory dynamics of lidocaine

**DOI:** 10.1007/s10266-021-00596-2

**Published:** 2021-03-05

**Authors:** Shoko Fujimori, Katsuhisa Sunada

**Affiliations:** Department of Dental Anesthesiology, The Nippon Dental University School of Life Dentistry at Tokyo, 1-9-20, Fujimi, Chiyoda-ku, Tokyo 102-8159 Japan

## Correction to: Odontology 10.1007/s10266-020-00585-x

In the original publication of the article, an unwanted asterisk was found in Fig. 3. The correct Fig. 3 is provided below.

**Fig. 3:**
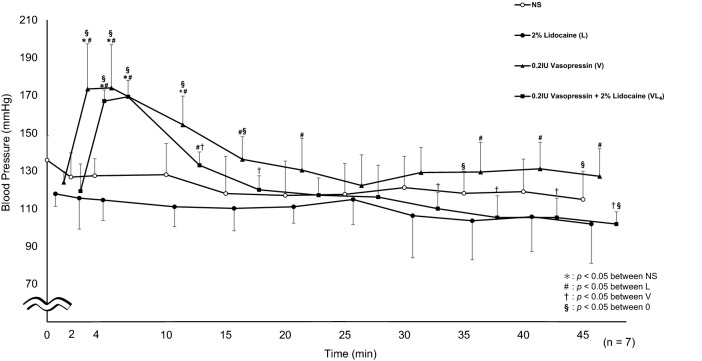
Systolic blood pressure. Significantly higher values were seen in VL4 at 2, 4, and 10 min (*p* < 0.0001, *p* < 0.0001, *p* = 0.0011, respectively) compared to L. VL4 was significantly elevated at 2, 4, and 45 min (*p* < 0.0001, *p* < 0.0001, *p* = 0.0192, respectively) compared to 0 min

The original article has been updated.

